# Data comparing the plasma levels of procollagen C-proteinase enhancer 1 (PCPE-1) in healthy individuals and liver fibrosis patients

**DOI:** 10.1016/j.dib.2017.08.047

**Published:** 2017-09-06

**Authors:** Eyal Hassoun, Mary Safrin, Eitan Wineman, Peretz Weiss, Efrat Kessler

**Affiliations:** a Maurice and Gabriela Goldschleger Eye Research Institute, Tel-Aviv University Sackler Faculty of Medicine, Sheba Medical Center, Tel-Hashomer, Ramat Gan 52621, Israel; b Liver Disease Center, Sheba Medical Center, Tel-Hashomer, Ramat Gan 52621, Israel

**Keywords:** Collagen biosynthesis, Liver fibrosis, biomarkers, ELISA

## Abstract

This article provides a protocol for determination of human procollagen C-proteinase enhancer 1 (PCPE-1) concentrations by ELISA. The inter-assay and intra-assay coefficients of variability are given and so are the average plasma concentrations of PCPE-1 in healthy (control) individuals and liver fibrosis patients.

**Specifications Table**

**Table:** 

Subject area	Biology, Biochemistry
More specific subject area	Plasma markers; Tissue Fibrosis
Type of data	Tables; Figures
How data was acquired	ELISA
Data format	Analyzed
Experimental factors	Measurement of PCPE-1 concentrations in human plasma using sandwich ELISA
Experimental features	A procedure for determination of human PCPE-1 concentrations by ELISA was developed using a specific monoclonal antibody for immobilization of human PCPE-1 and a polyclonal rabbit antibody against human PCPE-1 for its detection. PCPE-1 concentrations in plasma samples from healthy individuals and liver fibrosis patients were determined using this assay.
Data source location	Tel Aviv University, Tel Aviv, Israel
Data accessibility	Data is provided within this article

**Value of data**

• An ELISA method for determination of procollagen C-proteinase enhancer 1 (PCPE-1) concentrations in human plasma is described.
• The method can be used for determination of PCPE-1 concentrations in other body fluids.
• The data highlights the potential of PCPE-1 as a new non-invasive biomarker of liver fibrosis, which could be valuable clinically.

## 1 Data

**Fig. 1:**
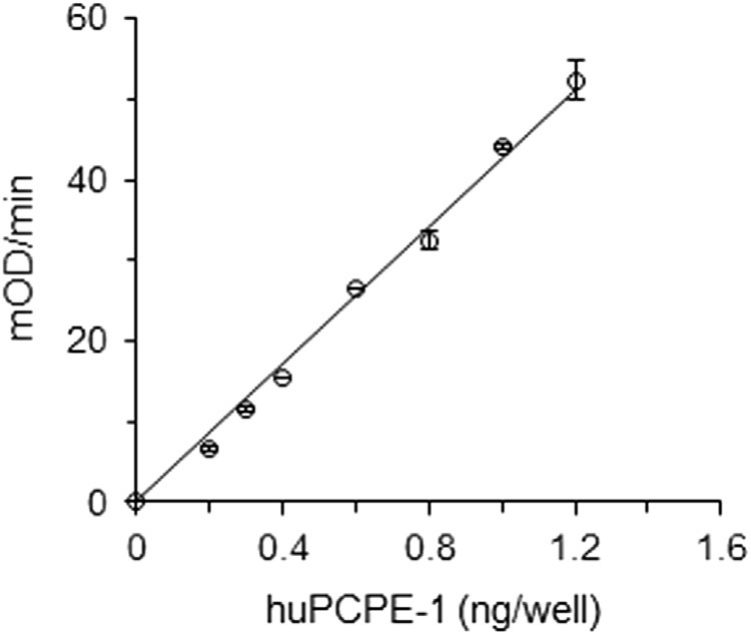
Standard curve for quantification of huPCPE-1 by sandwich ELISA. Increasing amounts of purified recombinant huPCPE-1 were adsorbed to wells pre-coated with a mouse monoclonal antibody to huPCPE-1. Bound PCPE-1 was detected using a rabbit polyclonal antibody to huPCPE-1 and quantified using an APA-conjugated goat anti rabbit IgG antibody. Each value represents mean±standard deviation (SD); *n*=2. mOD, Optical Density at 405 nm expressed in milli units.

**Fig. 2:**
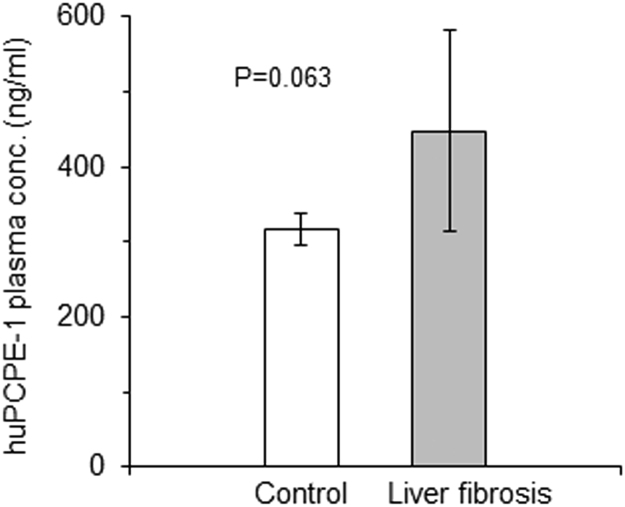
PCPE-1 plasma concentrations in healthy (control) subjects and liver fibrosis patients. Data from Table 1, Table 2 (presented as the mean±standard deviation; *n*=5).

The data includes two Figures and three Tables. Fig. 1 presents a calibration curve for determination of human PCPE-1 concentrations. Table 1, Table 2 provide the plasma concentrations of PCPE-1 in healthy individuals and liver fibrosis patients, respectively. Table 3 summarizes the clinical features of the patients. Fig. 2 compares the average plasma concentrations of PCPE-1 in healthy individuals and liver fibrosis patients. Supplemental Tables S1 and S2 provide raw data used to calculate the inter- and intra-assay coefficients of variability, respectively.

**Table 1:** Plasma concentrations of PCPE-1 in healthy (control) individuals.

Individual	Gender	Age	PCPE-1 plasma concentration (ng/ml±SD)
C1	F	31	312.7±16.5
C2	F	56	339.2±14.4
C3	M	52	317.8±35.1
C4	M	28	285.5±51.1
C5	M	35	329.1±44.4
Mean±SD			316.9±20.3^a^

a This value is practically identical to the previously reported [4] value of 305 ng/ml (median; obtained by Western blotting of human sera samples and quantification of PCPE-1 band intensity), supporting the validity of our ELISA. C, control (healthy); F, female; M, male; Age is expressed in years. *n*=8 (two dilutions, each measured in duplicates on two different days).

**Table 2:** Plasma concentrations of PCPE-1 in liver fibrosis patients.

Patient	Gender	Age	PCPE-1 plasma concentration (ng/ml±SD)
P1	F	59	342.3±26.9
P2	F	42	618.5±32.1
P3	M	56	433.3±24.7
P4	M	54	299.9±31.0
P5	M	64	546.8±30.1
Mean±SD			448.1±134.3

P, patient; F, female; M, male; Age is expressed in years. *n*=4 (two dilutions each measured in duplicates).

**Table 3:** Etiology, clinical features and MELD indexes of liver fibrosis patients.

Patient	Cause	Complications	Additional disorders	MELD
P1	HBV	Gastropathy, Controlled ascites	Hypertension, Obesity, Ovary cancer	2
P2	HBV	Esophageal varices	None	4
P3	HCV	Bleeding varices	Diabetes Mellitus Type 2	5
P4	HBV	Bleeding varices, Controlled ascites, Encephalopathy	Diabetes Mellitus Type 2	12
P5	HCV	None	Hypothyroidism	9

P, patient; HBV, hepatitis B virus; HCV, hepatitis C virus; MELD, Model for End-stage Liver Disease (a measure of mortality risk in patients with end-stage liver disease; http://www.mayoclinic.org/medical-professionals/model-end-stage-liver-disease).

## 2 Experimental design, materials and methods

### 2.1 Proteins and antibodies

Human recombinant PCPE-1 (huPCPE-1) was produced in 293-EBNA cells and purified from conditioned culture media as described [1], [2]. Monoclonal antibody 7A11/1 to huPCPE-1 was produced in our laboratory and is available commercially (Sigma, Santa Cruz). Rabbit polyclonal antibody to human recombinant PCPE-1 was prepared in our laboratory and its IgG fraction isolated from the serum using standard protocols. Similar polyclonal antibodies are available commercially (AssayPro, Proteintech etc.) and can be used instead. Alkaline phosphatase (APA) conjugated goat antibody to rabbit IgG, a monoclonal antibody against the Flag peptide (M2), and bovine serum albumin (RIA grade; cat # A7888) were from Sigma.

### 2.2 Experimental design

Experiments were approved by the Sheba Medical Center ethics committee (SMC-9650-12). Five liver cirrhosis patients at ages 42–64 years old and five healthy individuals at ages 28–56 years old were randomly selected. All of the participating individuals provided written informed consent.

### 2.3 Preparation of plasma samples

Blood was drown into plastic Citrate tubes (BD). After one to two hours at room temperature, the tubes were centrifuged (2000*g*; 15 min; room temperature) and plasma was transferred into Eppendorf tubes. Plasma samples were divided into aliquots and stored at −80 °C until use, avoiding repeated thawing and freezing.

### 2.4 Sandwich ELISA

The ELISA for human PCPE-1 was conducted as previously described [3] with the following modifications: (1) samples contained either known amounts of huPCPE-1 [1] (Fig. 1) or human plasma samples diluted in 5% BSA in PBS (blocking buffer); (2) wells were coated with monoclonal antibody 7A11/1 (5 μg/ml) to capture the antigen; (3) bound huPCPE-1 was detected using rabbit polyclonal antibody to huPCPE-1 (IgG fraction; 0.1 μg/ml); (4) APA-conjugated goat antibody to rabbit IgG (1:2,000) served for quantification of huPCPE-1. As a control, wells were coated with equivalent amounts of monoclonal antibody M2 (vs. the Flag peptide; instead of antibody 7A11/1), in which case, absorbance was identical to that of the blanks (uncoated wells or wells to which no PCPE-1 was added) namely, no PCPE-1 binding was evident.

### 2.5 Statistical analysis

Statistical significance was evaluated using two-tailed independent *t*-test. *P* value <0.05 was considered statistically significant.
