# Supplementary material for: Data comparing the plasma levels of procollagen C-proteinase enhancer 1 (PCPE-1) in healthy individuals and liver fibrosis patients
Source: Data Brief. 2017 Sep 6;14:777–81. doi: 10.1016/j.dib.2017.08.047 (PMC5602882; doi:10.1016/j.dib.2017.08.047)
Supplement: Supplementary file 2 — Supplementary material [file mmc2.docx]

Table S1. Determination of inter-assay coefficient of variability

| huPCPE-1 plasma concentration (ng/ml) | | | | | |
| --- | --- | --- | --- | --- | --- |
| Mean ± SD | 1:40 | | 1:20 | | Dilution |
|  | result 2 | Result 1 | Result 2 | Result 1 | assay # |
| 339.73 ± 3.92  333.23 ±14.83  330.72 ± 4.42  339.98 ± 1.56  334.45 ± 2.49  339.73 ± 6.23  326.95 ± 2.85  336.65 ± 4.56  340.82 ± 1.35  329.90 ± 7.20 | 341.99  344.20  331.16  339.62  336.79  340.27  329.06  341.17  339.93  337.71 | 338.70  317.48  324.76  342.27  335.12  334.77  325.85  336.58  339.32  327.49 | 344.34  319.80  329.82  340.16  335.62  334.18  322.83  329.27  342.83  319.26 | 333.89  351.43  337.16  337.89  330.27  349.71  330.07  339.56  341.22  335.12 | 1  2  3  4  5  6  7  8  9  10 |
| Mean of means 335.22  Mean of SDs 4.94  CV 0.02 | | | | | |

PCPE-1 concentrations in a plasma sample from the same healthy individual were determined on ten different days using a calibration curve created on the same day. Inter-assay coefficient of variability was derived as described (<http://www.poultry-health.com/library/serodiss/assayqc.htm>). SD, standard deviation; CV, coefficient of variability.
