# Supplementary material for: Data comparing the plasma levels of procollagen C-proteinase enhancer 1 (PCPE-1) in healthy individuals and liver fibrosis patients
Source: Data Brief. 2017 Sep 6;14:777–81. doi: 10.1016/j.dib.2017.08.047 (PMC5602882; doi:10.1016/j.dib.2017.08.047)
Supplement: Supplementary file 3 — Supplementary material [file mmc3.docx]

Table S2. Determination of intra-assay coefficient of variability

|  | huPCPE-1 plasma concentration (ng/ml) | | | | | | | |
| --- | --- | --- | --- | --- | --- | --- | --- | --- |
| Dilution | 1:20 | | | 1:40 | | Mean | SD | % CV |
| Sample # | Result 1 |  | Result 2 | Result 1 | Result 2 |  |  |  |
| C1  C2  C3  C4  C5 | 332.00  332.88  295.60  341.42  304.48 |  | 326.49  341.32  287.68  351.08  285.12 | 328.26  347.78  376.83  315.34  291.57 | 318.27  330.17  363.19  314.16  290.10 | 326.26  338.04  330.83  330.50  292.82 | 5.80  8.05  45.70  18.62  8.25 | 1.80  2.38  13.81  5.63  2.81 |
| P1  P2  P3  P4  P5 | 319.17  589.90  418.87  277.63  526.08 |  | 314.99  586.30  407.79  264.65  514.70 | 373.59  643.61  469.87  336.04  587.99 | 361.27  654.03  436.71  321.07  558.35 | 342.25  618.46  433.31  299.85  546.78 | 29.55  35.34  27.13  34.12  33.11 | 8.63  5.71  6.26  11.38  6.06 |
| Mean of % CV |  | | | | | | | 6.45 |

PCPE-1 concentrations in plasma samples from five healthy individuals and five liver fibrosis patients were determined on the same day. Mean values for C1-C5 differ slightly from those shown in Table 1 because the data in Table 1 are based on results of two sets of measurements (each performed on a different day; n=8) whereas the values presented here display results from one set of measurements (n=4). Intra-assay coefficient of variability was calculated as described (<http://www.poultryhealth.com/library/serodiss/assayqc.htm>). SD, standard deviation; CV, coefficient of variability. % CV is the ratio between the SD value and respective mean plasma concentration of PCPE-1, expressed as percentage.
